# Mature teratoma of the spinal cord in adults: An unusual case

**DOI:** 10.3892/ol.2013.1519

**Published:** 2013-08-08

**Authors:** YUAN LI, BO YANG, LAIJUN SONG, DONGMING YAN

**Affiliations:** Department of Neurosurgery, The First Affiliated Hospital of Zhengzhou University, Zhengzhou, Henan 450052, P.R. China

**Keywords:** intradural, intramedullary, spinal cord, teratoma, adult, case report

## Abstract

Intraspinal mature teratomas rarely occur in adults. The present study describes an unusual case of adult intradural mature teratoma, which was completely resected. A 22-year-old female presented with an intermittent pinching pain in the lower right shank that had lasted for three months. Magnetic resonance imaging (MRI) results indicated a multicystic mass extending from the T12 to L2 vertebrae, and the tumors were certified as teratomas by a histopathological examination. The level of pain experienced by the patient was improved following the surgery. The present study also compared the literature concerning adult intradural mature teratoma, summarized the basic clinical characteristics and theory of origin of adult intradural mature teratoma and reviewed the available treatments for this disease.

## Introduction

Teratomas are a type of multipotential cell tumor that contain a mixture of multiple germinal layers formed by normal organogenesis and reproductive tissues. Based on the degree of differentiation, teratomas may be classified as mature, immature or malignant (1). The incidence of spinal teratoma is rare; only 0.15–0.18% of spinal tumors have been classified as teratomas (2). In pediatric patients, ~5–10% of spinal tumors are intraspinal teratomas (3–5), however, the incidence in adult patients is significantly lower than that observed in children and infants (6–12).

Unlike in intraspinal teratomas in infants and children, the symptoms of these tumors in adult patients typically lack specific clinical features that, upon diagnosis, may cause confusion with other spinal tumors, such as schwannomas, which are more commonly observed in adult patients (13). At present, the mechanism of intraspinal teratoma formation and the prognosis of the disease following surgery have not yet been elucidated. Written informed consent was obtained from the patient.

## Case report

A 22-year-old previously healthy female presented with an intermittent pinching pain in the lower right shank that had lasted for three months, progressive lower right extremity weakness and instability while standing. The onset of the shank pain and weakness occurred without any obvious cause. After one month, the patient experienced a shooting pain from the right shank, which traveled towards the right groin, foot and thigh. The patient had not received spinal surgery or any other spinal procedures, and did not complain of lower extremity numbness or urinary incontinence. The physical examinations revealed that there were no motor or sensory deficits in either extremity, and no palpable midline spinal displacement. Upon neurological examination, the patient demonstrated normal physical reflexes and the pathological examinations revealed no cutaneous abnormalities or dermal sinus tracts. In addition, the routine laboratory examinations were normal.

**Figure 1:**
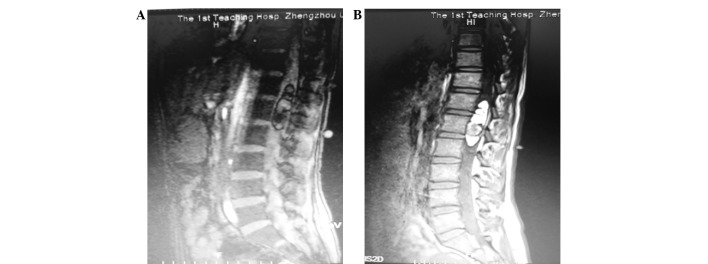
Axial magnetic resonance imaging (MRI) scans of the lesion. (A) Sigittal T1-weighted MRI contrast enhanced scan revealing an intramedullary mass located between T12 and L2 spinal cord levels. (B) Sigittal T2-weighted MRI image revealing a hyperintense, well-delineated intramedullary mass.

The magnetic resonance imaging (MRI) results revealed a lobulated, intradural, heterogeneous, 6.0×1.5×1.7-cm mass between T12 and L2 levels of the lumbosacral spine (Fig. 1). The lesion was located in the middle of the spinal canal and extruded the spinal cord, and could not be separated from the conus medullaris. No centrum erosion or other abnormalities were identified.

**Figure 2:**
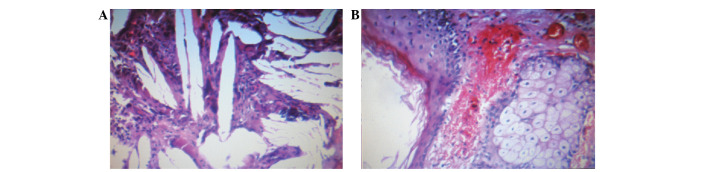
Histology of the excised mass. (A) Tumor consisting of stratified squamous epithelium, smooth muscle cells and adipose tissue (staining, hematoxylin and eosin; magnification, ×100). (B) Glial tissue cells and smooth muscle cells (staining, hematoxylin and eosin; magnification, ×200).

The patient underwent a total resection of the tumor by means of a T12-L1 laminectomy performed under a surgical microscope. Through an incision into the dura, three connected cystic tumors were observed. A portion of the mass was in contact with the medullary and conus medullaries, and a yellow, oval-shaped, fatty cyst extruded to the cauda equina where it had become inseparable. Following the incision into the tumor cyst wall located in the conus medullaris, a white fluid containing hair follicles and gray soft tumor tissue was observed. A histopathological examination of the excised mass revealed the presence of elements from multiple germ cell layers. Under a light microscope, numerous fatty cysts consisting of neuroepithelial and epithelial tissues were observed (Fig. 2). The final histopathological diagnosis was that of a mature cystic teratoma.

The prognosis of the patient, following surgery, was good. No further neurological deterioration was observed during the three-month follow-up period and the leg pain symptoms were relieved subsequent to the surgery.

## Discussion

Mature teratomas are a type of benign germ cell tumor, rarely observed in adult patients. The literature concerning adult intradural mature teratoma was reviewed from 1928 to the present date (14–25), and the relevant data from these cases are summarized in Table I.

The incidence of mature intraspinal teratomas in adults is rare, however, certain common features may be noted. Adult patients with mature intraspinal teratomas typically present with a delitescent onset. The literature revealed that, unlike in infants and children, intraspinal mature teratomas in adult patients were rarely observed with vertebral body anomalies or thoracolumbar spinal bifida (14,20,26–29). The main symptom endured by the adult patients included a numbness or weakness of the lower-extremities, occasionally accompanied by pain. Although the adult intraspinal teratoma patients commonly experienced a certain extent of neurological disorder, a decline of motor grade was not obvious (14,15,20,24–26,28,29). Compared with in the infants and children, the lesions in the adult patients were more localized. The tumors were predominantly located between the lower thoracic vertebrae and the conus medullaris level (30,31). The MRI images of the tumors were usually used as diagnostic evidence of an intraspinal mature teratoma. The morphological presentation varied in the MRI scans according to the location of the tumors. Intradural teratomas were commonly oval or lobulated heterogenous masses, whereas extradural teratomas were more commonly observed to be dumbbell-shaped. Cases of extradural teratoma are commonly accompanied with vertebral body misformation, while adult intradural teratomas are typically located beneath the dura, rarely invading the dura or vertebral body. The performance of a histopathological examination subsequent to surgery is the final analysis required to confirm the diagnosis of an intraspinal mature teratoma (10). Using light microscopy, histopathological slides of adult mature teratoma sections demonstrate multiple germinal tissue layers. The analysis of the literature revealed that in a number of cases, only two of the three germinal layers were observable; this may have been due to the fact that the derivatives of one or two of the layers had grown over the others (9,16). Several tumor markers, including serum β-human chorionic gonadotropin (β-hCG) and α-fetoprotien (AFP), were applied for the diagnosis and prognosis of recurrences of sacrococcygeal teratoma; however, this application was limited in the mature teratomas as the recurrence may have originated from non-secreting parts of the previous lesion (32).

There are two dominant theories regarding the origin of intraspinal teratomas. The first is the dysembryogenic theory, and the second is the misplaced germ cell theory (33,34). The dysembryogenic theory indicates that spinal teratomas arise from pluripotent cells, and that in a locally disturbed developmental environment, these pluripotent cells differentiate chaotically. When such disordered development occurs in a primitive streak or a caudal cell mass, a spinal teratoma forms (21,35). The misplaced germ cell theory suggests that certain pluripotent primodial germ cells of the neural tube are misplaced during migration from the yolk sac to the gonad, thus resulting in spinal teratoma formation (34).

There is evidence to support the rationale of each theory. To the best of our knowledge, dysraphic malformations are considered to support the dysembryogenic theory. The tridermal anomaly is the primary event of the disordered development of pluripotent cells in the spine, which is likely to further affect the spinal closure (21). Occurrence of a neurenteric cyst without dysraphism also supports the dysembryogenic theory (36). The explanation of isolated teratomas that are considered to have arisen by this theory is frequently questioned. The most common location for a spinal teratoma is between the lower thoracic vertebrae and the conus medullaris, which is adjacent to the caudal cell mass. This supports the theory that teratomas originate from the stochastic misplacement of a pluripotent germ cell from the caudal cell mass (30). As the caudal cell mass originates from Hensen’s node, the possibility that teratomas may arise from the chaotic differentiation of pluripotent cells in Hensen’s node during caudal elongation is also a plausible theory. One study that isolated three stem cell lines from sacrococcygeal teratomas also suggested that a caudal cell mass was the likely origin of teratomas (37).

In adult intraspinal teratomas, which rarely present with significant dysraphism, the misplaced germ cell theory is likely to be more feasible (10,31,33,38,39). Studies focusing on 22 cases of germ cell tumors located in the spinal cord support this modified theory (40–42). In addition, the presence of ectopic primordial germ cells in the caudal cell mass has also suggested that intraspinal teratoma may result from misplaced germ cells (43). According to this theory, the disruption of the developmental field and dysraphism may be explained by the growth of the teratoma (44).

The primary treatment for teratomas is surgery, which may also be applied to mature intraspinal teratomas. An epidemiological study of spinal teratomas revealed that the recurrence rates for complete and gross resection were extremely similar (9 and 11%, respectively) (32), and that the nature of mature teratomas was relatively benign. Therefore, the dominant guide for intraspinal teratoma surgery did not recommend radical resection (9,10,45). In a study of teratomatous cysts of the spinal canal, the wall of the cyst was in intimate contact with the adjacent neural tissue in almost half of cases. This would render radical resection more difficult and affect the patient’s prognosis (16). In the present case, complete resection was achieved without the injury to adjacent neural tissues, and thus, no further neurological defects were observed following the surgery. The determination of whether the residual remnants of a lesion may regrow to form a new tumor requires long-term follow-up. Due to the extremely low incidence of adult mature spinal teratoma and the limited knowledge of the disease, adjuvant therapy for such teratomas remains controversial (32). It is commonly accepted that post-operative adjuvant therapy ought to depend on the pathological examination. The application of radiotherapy is justified when malignant histological features or germ cell elements have been confirmed. Following surgery, patients should be followed up with serial MRI examinations and the potential side-effects of any radiotherapy should be considered (31). The efficacy of chemotherapy as a treatment for this disease has not been demonstrated (32).

Mature intradural teratomas in adults are rare, with few accompanying spinal anomalies. The currently preferred theory of origin of the disease is the misplaced germ cell theory. A resection of the tumor is the primary treatment methodology for adult patients, as the nature of the tumor is relatively benign and the recurrence ratio is low, even following gross resection. However, radiosurgery is not recommended.

## Figures and Tables

**Table I:** Review of adult intradural teratoma cases reported in the literature, from 1928 to the present day.

First author/s, year	No. of cases	Gender	Mean age (years)	Location	Associate abnormal	Resection
Kubie and Fulton, 1928	1	F	27.0	C3–C4	Absent	Incomplete
Hosoi, 1931	1	M	24.0	L2–L3	L5–S1 spina bifida	Incomplete
Sullivan, 1948	1	F	32.0	L1–L3	Absent	Complete
Bakay, 1956	1	F	65.0	L1–L2	L1&L2 vertebral body fusion body fusion	Incomplete
Sloof *et al*, 1964	1	M	20.0	L1	Absent	Complete
Rewcastle and Francoeur, 1964	1	F	34.0	T10	Absent	Incomplete
Hansebout and Betrand, 1965	1	M	47.0	L1–L3	Absent	Complete
Enestom and Von Essen, 1977	1	M	36.0	T11-L1	Absent	Incomplete
Rosenbaum *et al*, 1978	1	M	49.0	T9	Absent	Complete
Garrison and Kasdon, 1980	1	M	23.0	L2	Absent	Complete
Padovani *et al*, 1983	1	F	33.0	T12-L1	Absent	Complete
Pelissou-Guyotat *et al*, 1988	1	M	33.0	L4	L4 spina bifida occulta	Complete
Nicoletti *et al*, 1994	1	M	47.0	Conus medullaris	Conus medullaris caudal exophy	Incomplete
Caruso *et al*, 1996	1	M	41.0	Conus medullaris	Absent	Complete
Al-Sarraj *et al*, 1998	1	M	35.0	Conus medullaris	Absent	Incomplete
Poeze *et al*, 1999	1	M	23.0	T12-L1	Absent	Incomplete
Fan *et al*, 2001	1	F	43.0	L2	Absent	Complete
Nonomura *et al*, 2002	2	1F, 1M	44.5	1T12-L1, 2T12-L2	Absent	Incomplete
Hejazi and Witzmann, 2003	2	1F, 1M	32.5	1T11-L3, 2 L2–L4	Absent	Complete
Fernandez-Cornejo *et al*, 2004	1	M	43.0	L1–L2	Absent	Complete
Ak *et al*, 2006	1	F	43.0	C2–C3	C3 spina bifida, C5 level nodule	Complete
Makary *et al*, 2007	1	F	46.0	C1–C2	C1–C2 dysraphic congenital spinal malformations	Complete
Biswas *et al*, 2009	1	M	28.0	L2–L4	Absent	Complete
Ghostine *et al*, 2009	1	F	65.0	C1–C2	Absent	Incomplete
Ijiri-Kosei *et al*, 2009	1	F	68.0	L1–L2	Absent	Complete
Present case	1	F	22.0	T12-L2	Absent	Complete

M, male; F, female; C, cervical; T, thoracic; L, lumbar.
